# A Topographical Analysis of Encephalocele Locations: Generation of a Standardised Atlas and Cluster Analysis

**DOI:** 10.1007/s00381-023-05883-7

**Published:** 2023-03-10

**Authors:** Vejay N. Vakharia, Sebastien Toescu, Andrew J. Copp, Dominic N.P. Thompson

**Affiliations:** 1 Department of Neurosurgery, Great Ormond Street Hospital for Children NHS Foundation Trust, London, United Kingdom; 2 Developmental Biology & Cancer Department, UCL Great Ormond Street Institute of Child Health, London, United Kingdom

## Abstract

**Objective:**

Encephaloceles are considered to result from defects in the developing skull through which meninges, and potentially brain tissue, herniate. The pathological mechanism underlying this process is incompletely understood. We aimed to describe the location of encephaloceles through the generation of a group atlas to determine whether they occur at random sites or clusters within distinct anatomical regions.

**Methods:**

Patients diagnosed with cranial encephaloceles or meningoceles were identified from a prospectively maintained database between 1984 and 2021. Images were transformed to atlas space using non-linear registration. The bone defect, encephalocele and herniated brain contents were manually segmented allowing for a 3-dimensional heat map of encephalocele locations to be generated. The centroids of the bone defects were clustered utilising a K-mean clustering machine learning algorithm in which the elbow method was used to identify the optimal number of clusters.

**Results:**

Of the 124 patients identified, 55 had volumetric imaging in the form of MRI (48/55) or CT (7/55) that could be used for atlas generation. Median encephalocele volume was 14704 [IQR 3655-86746] mm^3^ and the median surface area of the skull defect was 679 [IQR 374-765] mm^2^. Brain herniation into the encephalocele was found in 45% (25/55) with a median volume of 7433 [IQR 3123-14237] mm^3^. Application of the elbow method revealed 3 discrete clusters: 1) Anterior skull base (22%; 12/55), 2) Parieto-occipital junction (45%; 25/55) and 3) Peri-torcular (33%; 18/55). Cluster analysis revealed no correlation between the location of the encephalocele with gender [χ^2^ (2, n=91) = 3.86, p = 0.15]. Compared to expected population frequencies, encephaloceles were relatively more common in Black, Asian and Other compared to White ethnicities. A falcine sinus was identified in 51% (28/55) of cases. Falcine sinuses were more common [χ^2^ (2, n=55) = 6.09, p = 0.05] whilst brain herniation was less common [χ^2^ (2, n=55) =.16.24, p < 0.0003] in the parieto-occipital location.

**Conclusion:**

This analysis revealed three predominant clusters for the location of encephaloceles, with the parieto-occipital junction being the most common. The stereotypic location of encephaloceles into anatomically distinct clusters and the coexistence of distinct venous malformations at certain sites suggests that their location is not random and raises the possibility of distinct pathogenic mechanisms unique to each of these regions.

## 1 Introduction

Encephaloceles are part of the spectrum of neural tube defects (NTDs) and have an estimated prevalence of 1-5 per 10,000 live births worldwide and account for 10-20% of all craniospinal dysraphisms^1–4^. Encephaloceles are associated with numerous genetic syndromes including Walker-Warburg, Knobloch, Meckel, Fraser and Robert syndrome as well trisomies 13, 18 and 21, yet a unifying pathogenic mechanism remains elusive^5^. Environmental factors including hyperthermia, maternal malnutrition and aflatoxins are also associated with higher rates of encephalocele^6,7^. Occipital encephaloceles are more commonly associated with those of White ethnicities whilst anterior skull base encephaloceles are seen with greater frequency in Southeast Asian and African populations^7,8^. Children with encephaloceles can suffer significant morbidity as a result of seizures, learning disability, hydrocephalus and other associated congenital malformations^9,10^. Prenatal ultrasonography and MRI imaging have increased the detection of encephaloceles in utero and, in cases where the overlying skin is deficient, early surgical repair is usually recommended, to prevent infection, cerebrospinal fluid leak and progressive brain herniation^11^. Intrauterine repair has also been reported with a suggestion that this may improve cognitive outcomes ^12^. Prolapsed brain parenchyma within encephaloceles is usually dysplastic and non-functional but can present a unique surgical challenge if associated with essential vascular structures such as dural venous sinuses^13^.

The classical teaching that has been propagated in the medical literature since von Recklinghausen in 1886^14^, suggests that encephaloceles are NTDs that arise from a failure of closure of the anterior neuropore which typically occurs between the 3^rd^ and 4^th^ weeks of gestion^15^. However, careful analysis of human embryos^5^ and studies of multiple mouse NTD models^16^, have demonstrated that failure of neural tube closure in the cranial region leads to anencephaly, not encephalocele. Instead, post-neurulation pathogenesis, in which covering layers become deficient leading to herniation of the closed cranial neural tube, appears most likely^16,17^.

Through the generation of a group atlas, we sought to investigate whether encephaloceles in humans occur randomly or can be topographically localised to specific locations within the skull. We also applied clustering techniques to identify whether the clusters exhibit different characteristics with regards to gender, ethnicity, associated venous abnormalities, the surface area of the bone defect, brain herniation and volume of the encephalocele.

## 2 Methods

Approval for this study was granted by the Great Ormond Street Hospital for Children NHS Foundation Trust, Research and Development committee: R&D no.: 22DD04.

A prospectively maintained operative database from Great Ormond Street Hospital was searched to identify patients that had undergone surgical management of an encephalocele between 1984 and 2021.

Demographic information was derived from the electronic hospital records. Ethnicity data were categorised based on the English government recommended classification used in the U.K. 2011 census (https://www.ons.gov.uk/census/2011census). This included 18 ethnic groups which we subsequently assigned to 4 categories (Table 1). The 2011 census was also used to derive comparative population ethnicity data.

In cases where both MRI and CT were available, the MRI scan was the imaging modality of choice for segmentation of the encephalocele and extent of brain herniation, whilst the CT scan was used to delineate the extent of the bony defect.

**Figure 1:**
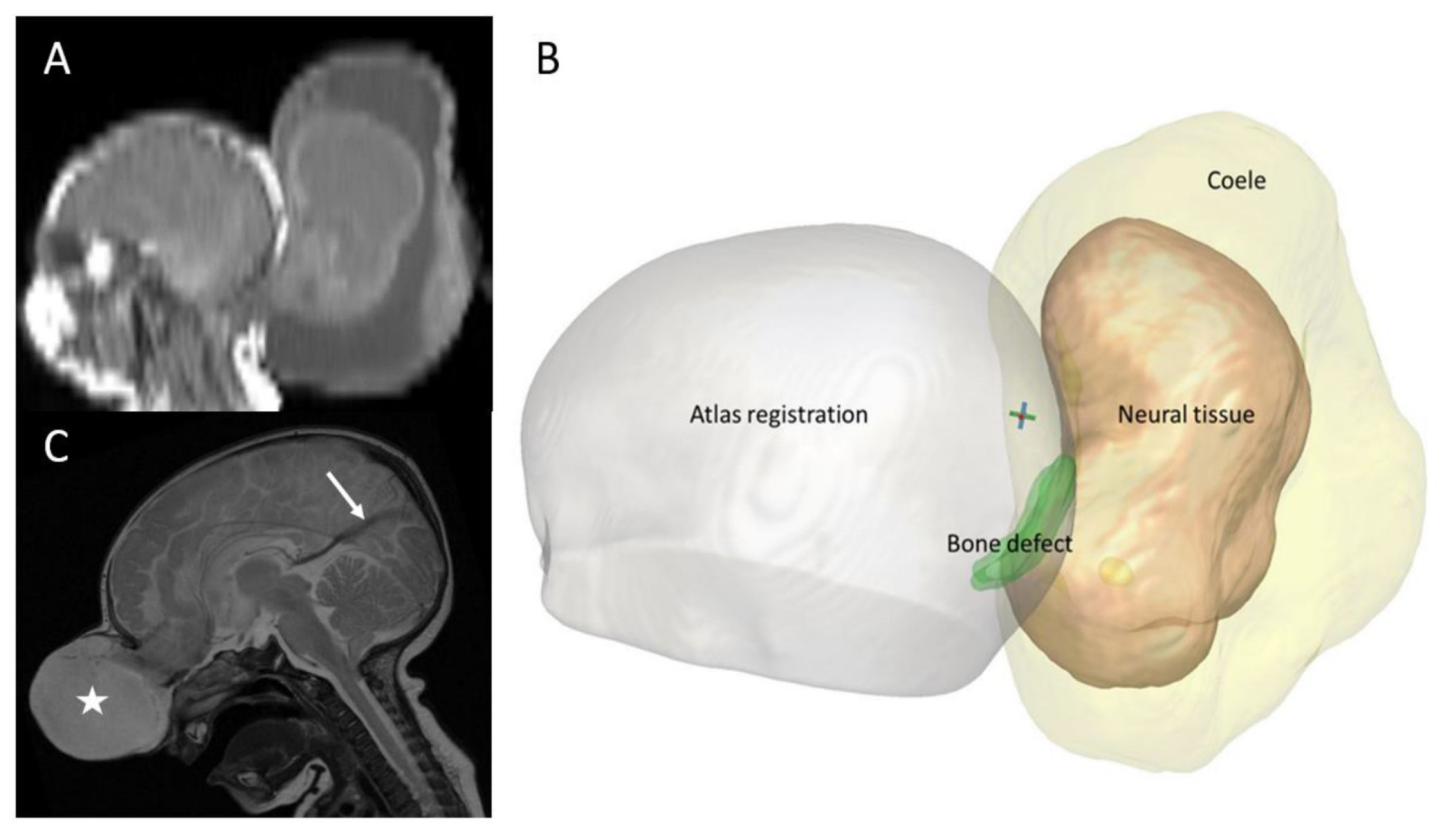
Example dataset findings. Example patient, A) post-natal sagittal MRI depicting a large peri-torcular encephalocele, B) 3D volumetric segmentation showing the bone defect (green), neural tissue (beige) and total encephalocele (yellow) after non-linear transformation to MNI-atlas space. C) Sagittal MRI image from a different patient with a falcine venous sinus (arrow) associated with an anterior skull base encephalocele (star).

All pre-operative imaging was registered to an age-appropriate atlas space, which is a composite template image generated from control data derived from the Montreal Neurological Institute (MNI) 11-14 month asymmetric set, utilising the ‘NiftiReg F3D’ non-linear transformation algorithm^18^. The surface area of the bone defect (mm^2^), the volume of the herniated brain (mm^3^) and total encephalocele volume (mm^3^) was then derived from manual segmentation (Figure 1).

The segmentation of the bone defects was combined to generate a heat map representing the topographical overlap between patients. The morphological centre (centroid) of the 3-dimensional bone defect was calculated and submitted to a K-means cluster analysis utilising custom scripts incorporating Python (3.9.7) libraries. The analysis was run repeatedly with cluster sizes varying from 1 to 10 until a stable solution was found to avoid inconsistencies that may result from random initialisation. The within-cluster sum of squares (WCSS), a surrogate marker of variance, was then calculated for each of the 10 cluster sizes and plotted graphically. The elbow method was applied to determine the optimal number of clusters which was then subsequently applied to the bone defect centroids. Further explanation of the clustering method is provided in the supplementary materials.

Correlations between cluster location and gender, ethnicity, bone defect surface area, brain volume and encephalocele volume were calculated. Statistical significance was determined through cross-tabulation of contingency tables and calculation of the Chi-squared statistic after correcting for multiple comparisons. A log transformation was applied to encephalocele and brain volume herniation to account for outliers and consequent right skew of the data.

## 3 Results

A total of 122 patients with encephaloceles were identified from the operative database. Of these, ethnicity information was disclosed in 96% (117/122), anatomical location of the encephalocele was identified in 73% (89/122) and pre-operative volumetric imaging suitable for the quantitative analysis was available for 45% (55/122) in the form of MRI 87% (48/55) and CT 13% (7/55). (Table 2 and Supplementary materials Figure 1)

### 3.1 Atlas generation

**Figure 2:**
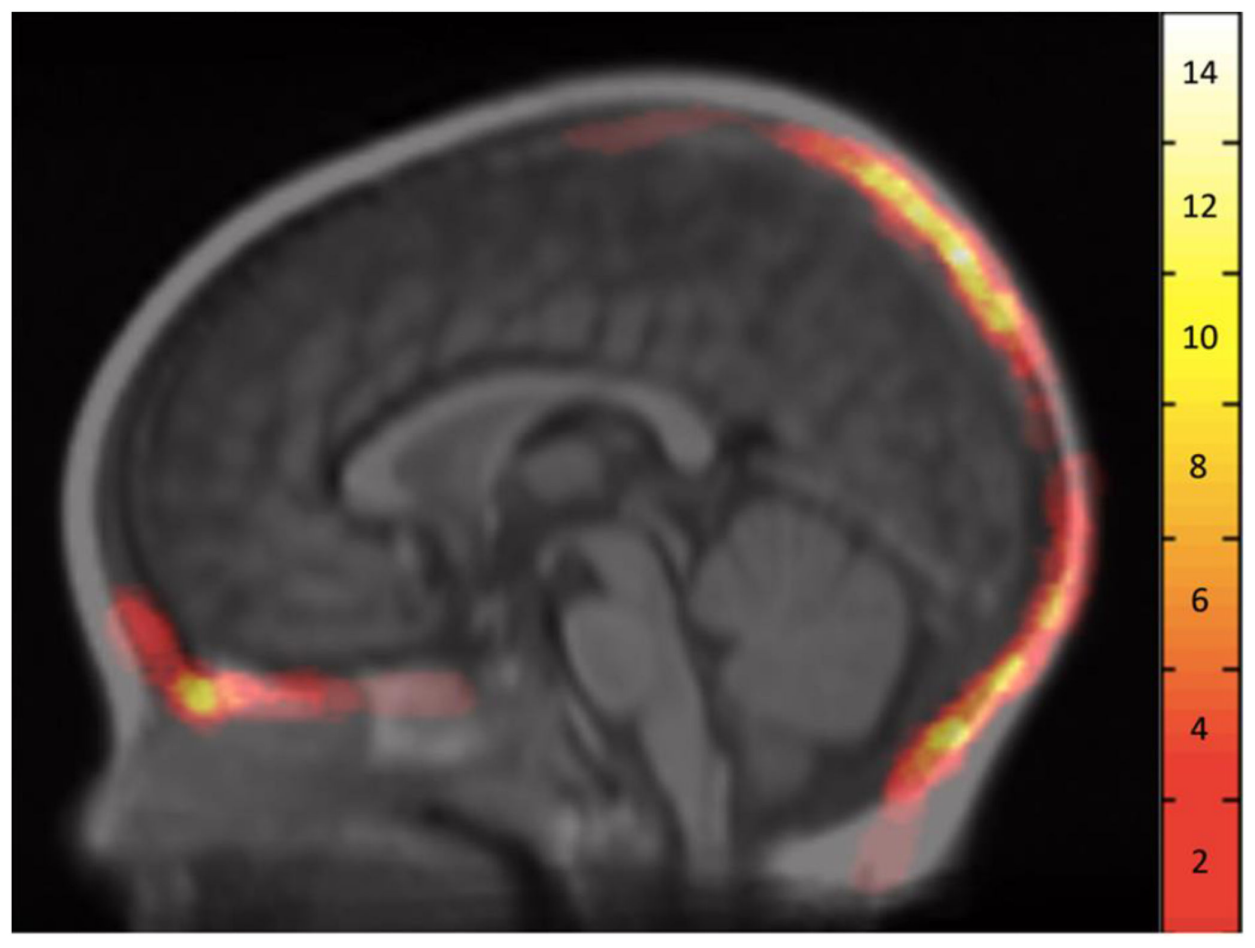
Atlas Space Heat Map of Bone Defect Overlap. Heat map generated through the overlap of bone defects in MNI (11-14 asymmetric atlas) space shown at the mid-sagittal plane. Note: MatLab Hot heat map applied with the colour bar to the right showing the number of subjects with overlapping bone defects.

After non-linear registration of the individual patient scans to the MNI atlas space, the volumetric representations of the bone defects were used to create a topographical heat map (Figure 2).

This represents the anatomical regions in which bone defect voxels overlap at a group level. Regions of greatest overlap, depicted in Figure 2 as white regions, were found at the anterior skull base in the vicinity of the foramen caecum, parieto-occipital junction and the peri-torcular locations.

### 3.2 Cluster analysis

Further quantitative analysis was undertaken through the application of a K-mean clustering algorithm. Unlike the topographical heat map where the entire extent of the bone defect was represented, the K-means clustering algorithm utilised the centroid of the bone defect expressed as a 3-dimensional coordinate. Given that all bone defects included the midline the X-coordinate, representing the medial-to-lateral component, was removed.

**Figure 3:**
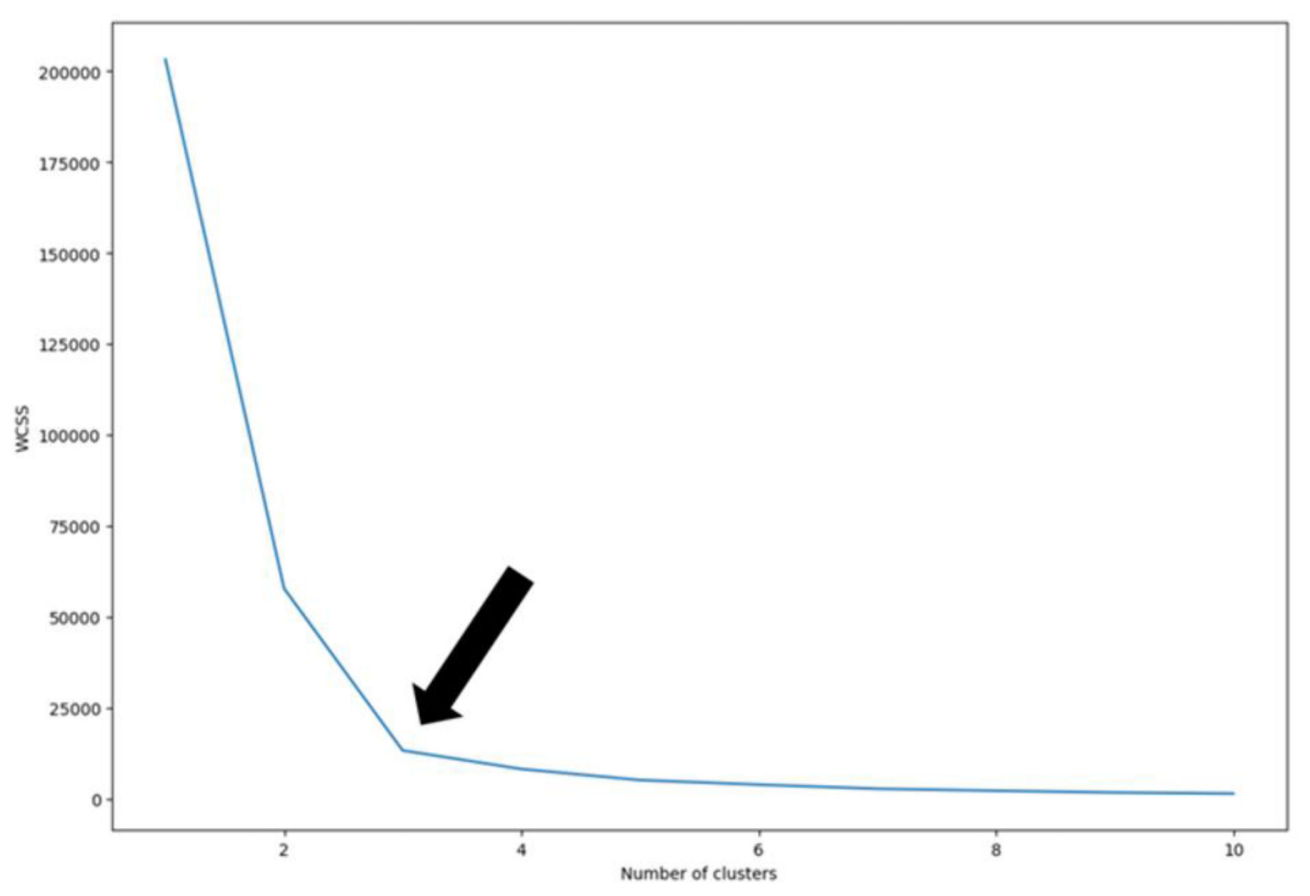
Demonstration of the Elbow Method. Demonstration of the elbow method using the within-cluster sum of squares (WCSS) as a quantitative representation of variance between individual bone defect centroids and the cluster centroid. When calculated for 1-10 clusters (K = 1-10), this demonstrates the fewest clusters resulting in the greatest reduction in WCSS i.e., ‘the elbow’ is 3 (black arrow).

This K-mean clustering algorithm requires the number of clusters (K) to be pre-defined. To determine the optimal number of clusters the ‘Elbow method’ was applied. The algorithm was applied with the number of clusters ranging from 1-10 and the within-cluster sum of squares (WCSS) was calculated for each. The smallest number of clusters with the lowest WCSS represents the elbow and signifies the optimal number of clusters as any further increase in the number of clusters does not result in any meaningful reduction in the WCSS ^19^. This suggests that the centroids of the bone defects can be optimally clustered into 3 discrete regions. (Figure 3)

**Figure 4:**
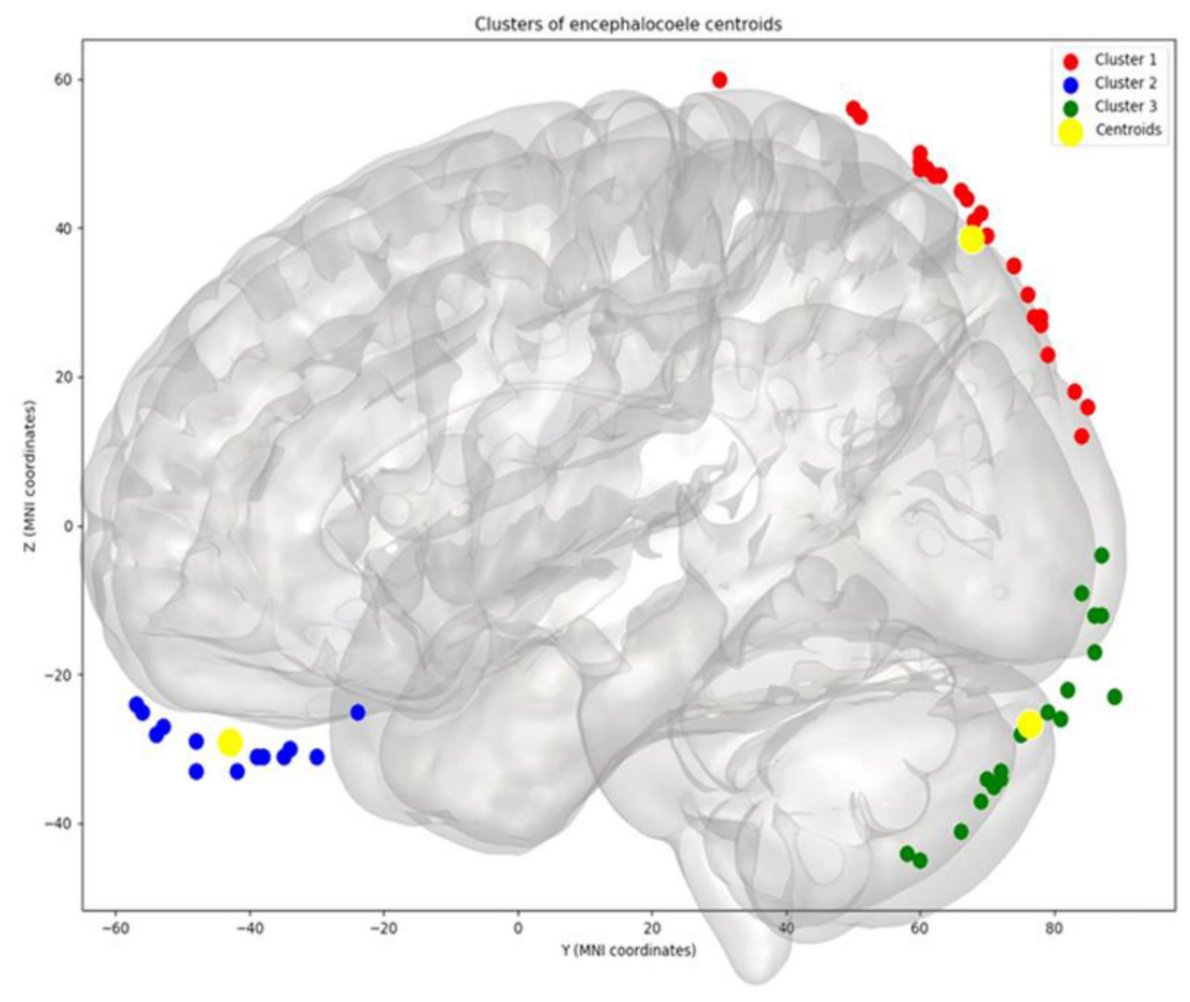
Results derived from Clustering (K=3). Application of the K-means clustering algorithm with K=3 revealed centroids of the bone defects to be classified into Cluster 1: parieto-occipital junction (red dots), Cluster 2: anterior skull base (blue dots) and Cluster 3: peri-torcular (green dots). The centroids of each cluster are also depicted (yellow dots).

Application of K=3 to the group data allowed for the bone defect centroids to be separated into three distinct clusters: 1) Anterior skull base 22% (12/55), 2) Parieto-occipital junction 45% (25/55) and 3) Peri-torcular 33% (18/55). (Figure 4)

### 3.3 Assigned cluster characteristics

**Figure 5:**
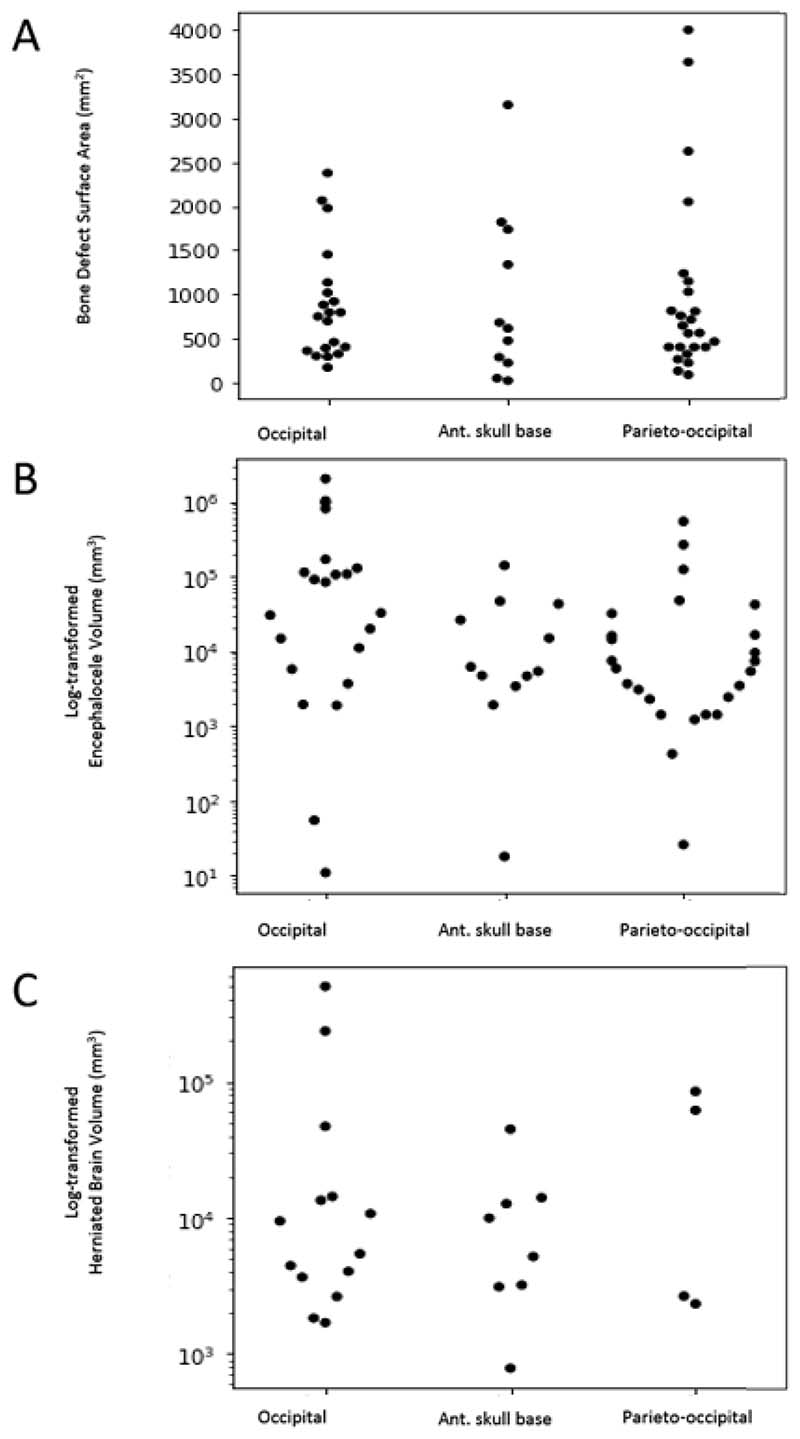
Summary of volumetric encephalocele analysis based on assigned clusters. Scatter plots demonstrating: A) Bone defect surface area (mm^2^), B) Encephalocele volume (mm^3^) and C) Herniated brain volume (mm^3^) distributions, based on the assigned clusters. Note, log transformations were applied to encephalocele and brain herniation volumes to account for outliers resulting in a right skew of the data.

Based on the anatomical cluster assignments derived from the K-means clustering analysis we were able to assign 91 patients from the entire cohort to clusters: peri-torcular 43% (39/91), parieto-occipital 35% (32/91) and anterior skull base 23% (21/91). We then sought to determine if there was any correlation between ethnicity, gender, the surface area of the bone defect (cm^2^), the volume of the herniated brain (cm^3^) and total encephalocele volume (cm^3^) (Figure 5).

Of the 91 patients with encephaloceles assigned to clusters, ethnicity information was available for 89. Based on Census data from 2011 in England the population ethnicity distribution was 86% White, 7.5% Asian, 3.3% Black and 1% Other. Based on our cohort we identified the ethnicity distribution of encephaloceles to be 52% (61/117) White, 16% (19/117) Asian, 18% (21/117)) Black and 14% (16/117) Other. Compared to the expected frequency from the Census data these results reveal a significantly greater frequency of encephaloceles in Asian [χ^2^ (2, n=54) = 15.11, p = <0.01], Black [χ^2^ (2, n=61) = 10.90, p = <0.05] and Other ethnicities [χ^2^ (2, n=53) = 17.98, p = <0.003] compared to White ethnicities (Table 3).

Herniation of brain contents into the sac was present in 47% (26/55) of the cohort overall with a median volume of 7433 [IQR 3123-14237] mm^3^ and was comparatively less frequent in the parieto-occipital compared to the anterior skull base and peri-torcular clusters [χ^2^ (2, n=55) = 16.24, p = <0.0003]. Encephaloceles were associated with female gender in 57% (70/122) of the cohort with a trend toward a greater incidence in the peri-torcular cluster (25 female:14 Male) but this failed to achieve significance [χ^2^ (2, n=89) = 3.86, p = 0.15].

The median encephalocele volume was 14704 [IQR 3655-86746] mm^3^ and the median surface area of the skull defect was 679 [IQR 374-765] mm^2^. Comparison of the log-transformed encephalocele volume between assigned clusters revealed that peri-torcular encephaloceles were the greatest in size (ANOVA test statistic=7.26, p= 0.002). There was no difference in the surface area (mm^2^) of the bone defects (Kruskal Wallis H-test statistic 0.24, p = 0.89) and the log-transformed herniated brain volume (ANOVA test statistic=0.89, p-value=0.42) between assigned clusters.

A falcine venous sinus was identified in 53% (29/55) of the cohort and was more commonly associated with a parieto-occipital encephalocele [χ^2^ (2, n=55) = 6.09, p = 0.05].

## 4 Discussion

Encephaloceles are characterised by defects in the skull through which the meninges and brain tissue herniate ^16^. The subsequent morbidity is related to the site and volume of the encephalocele, eloquence of the herniated brain tissue and associated congenital abnormalities^20^. Immediate surgical closure of large defects, especially when the overlying skin is defective, can prevent secondary infection; despite this, children often suffer severe lifelong neurological dysfunction as a result of epilepsy, hydrocephalus and learning disability with a mortality rate of around 1 in 3^3,21^. Much of our understanding of neural tube closure and the pathological mechanisms underlying subsequent defects are derived from chick and mouse models, yet comparatively little is known about human embryos^15^.

In this study, we aimed to determine if there are stereotyped anatomical regions in which encephaloceles arise in humans. We employ neuroimaging techniques to generate an atlas of bone defects associated with encephaloceles that have undergone surgical treatment in our unit since 1984. Registration of volumetric imaging to an age-appropriate atlas space allows the topographical relationship of the skull defects to be co-localised as well as the surface area of the bone defects, encephalocele and herniated brain volumes to be corrected for head size due to varying degrees of microcephaly. The morphological centre of the bone defect was calculated and subjected to a K-means clustering analysis which identified 3 discrete sites of encephalocele origin: 1) Anterior skull base, 2) Parieto-occipital junction and 3) Peri-torcular.

A previous classification of encephaloceles derived from cadaveric dissection of 12 patients was based on the anatomical location and categorised these into 1) Occipital, 2) Cranial vault, 3) Fronto-ethmoidal, 4) Basal and 5) Cranioschisis^22^. This system, however, was initially conceived to inform surgical approaches for repairing lesions. Since its inception in 1972, surgical techniques have advanced significantly, especially with the increased use of endoscopes to repair defects of the anterior skull base^23^. The system is also purely anatomical and does not provide information regarding frequency, associated cranial malformations or convergence towards specific anatomical locations, which may allow inferences to be made regarding possible pathogenic mechanisms. In our series, we did not observe any encephaloceles arising through the frontal region. In accordance with other similar reports from Western populations^21^, the peri-torcular location, also referred to as sub-torcular or occipital encephaloceles in the literature, was most frequent, representing 42% of the entire cohort and 45% of those included in the volumetric analysis. Previous studies have shown that anterior skull base encephaloceles are more common in South Asian populations, but it is still unclear whether this results from an underlying genetic susceptibility or exposure to environmental factors in early pregnancy^7^.

The notion suggested by von Recklinghausen in 1886^24^, that encephaloceles arise as a result of abnormal primary neurulation, specifically failure of anterior neuropore closure, is still propagated throughout the medical literature^25^. Primary neurulation is the process in which the neural plate bends so that its lateral aspects become elevated, eventually fusing in the dorsal midline to form the neural tube ^15^. This process begins at the hindbrain/cervical boundary and propagates into the cranial region and simultaneously along the future spine. Careful studies of human embryos that exhibit normal or abnormal cranial neural tube closure have concluded that faulty neuropore closure results in the open defect anencephaly^26,27^. This is consistent with the origin of open spinal NTDs (e.g. myelomeningocele) from faulty spinal neuropore closure. Studies of many mouse NTD models confirm that faulty neuropore closure results in exencephaly/anencephaly in the cranial region and open spina bifida in the caudal region^5^. Hence, the concept that encephalocele originates from faulty neural tube closure receives no support from the actual analysis of human and mouse embryos. Indeed, a recent study of mouse embryos in which the *Rac1* gene was conditionally deleted in the non-neural ectoderm, revealed that exencephaly and encephalocele are alternative cranial outcomes in individual embryos with this genetic alteration^16^. Importantly, exencephaly was shown to arise from faulty cranial neural tube closure, whereas occipito-parietal encephalocele arose post-neurulation, through herniation of the closed neural tube through a defect in the covering ectodermal layer. Further suggestions of a post-neurulation mechanism in humans come from the findings of both parietal and occipital encephaloceles within the same patients as distinct entities suggesting two focal regions in the ectoderm may have become deficient ^28–30^. The balance of evidence, therefore, favours a post-neurulation origin of encephalocele in both humans and mice.

The question then arises as to the primary tissue of origin of encephaloceles. Is the initiating lesion in the developing brain, skull or another tissue? As found in the present study, a skull opening is invariably present in encephalocele, forming the location of the meningeal/brain herniation. While a primary skull developmental defect is an attractive concept, it is complicated by the fact that the skull has a dual developmental origin: the frontal region and facial skeleton arise from neural crest cells, which emigrate from the neural tube and also give rise to the meninges, whereas the parieto-occipital region arises from cranial mesoderm. The skull base has a similar dual origin^31^. Hence, a primary skull lesion would need to involve different embryonic ‘germ layers’ at different encephalocele locations. In the Rac1 mouse model of occipito-parietal encephalocele^16^, a skull opening was present but this arose only after brain herniation was well established. In this case, the initiating lesion appeared to be a rupture of the non-neural ectoderm, to which the *Rac1* gene deletion had been targeted. It remains to be determined whether other instances of encephalocele will prove to have a comparable developmental origin.

In our study, brain herniation into the encephalocele sac occurred in almost half of the cases, but this was least likely in the parieto-occipital junction cluster, in keeping with clinical reports of preferential atretic encephaloceles at this location^32^. Of interest, an associated venous abnormality, specifically a falcine sinus, was associated with encephaloceles in all locations but was more frequent within the parieto-occipital location. The finding of a falcine sinus in patients with remote encephaloceles, such as in the anterior skull base, suggests that this is not simply due to the redirection of venous outflow secondary to the direct mechanical effect of the encephalocele. A falcine sinus is also seen in patients with vein of Galen malformations or lack of a straight sinus and represents a connection between the internal cerebral veins or vein of Galen and the superior sagittal sinus. In human embryos, the precursor to the superior sagittal sinus is known as the superior sagittal plexus, which forms when large tributaries of the anterior and middle plexuses merge in the midline^33^. This process begins at the 14 mm embryo length and is established by the 50 mm length stage. The deep venous system drains into the superior sagittal plexus through a primitive straight sinus that is discernible in the 20 mm length embryo. The internal cerebral veins on the other hand fuse to form the vein of Galen by the 80 mm stage. These findings suggest that in humans the defect in the ectomesoderm may be present before the 20 mm stage thus preventing the straight sinus from developing.

Peri-torcular encephaloceles were associated with the largest overall volume although the reasons for this remain unclear^11,20^. Previous human studies have also suggested an increased incidence in females ^17^. In our cohort we found a trend favouring encephaloceles in female patients, but this failed to reach statistical significance. Compared with White ethnicities we found a higher incidence of encephaloceles in Black, Asian and Other (Arab) ethnicities than expected from the population distribution of the United Kingdom derived from national census data^2,8^. This finding is likely to be skewed due to the international referral pattern to our institution. Nevertheless, patients from Black ethnicities trended towards a higher incidence of occipital encephaloceles whilst Arab ethnicities demonstrated more parieto-occipital encephaloceles.

Limitations of the study:

The most notable limitation is that the study is retrospect in nature. Consequently, volumetric imaging data sets of sufficient quality to contribute to atlas generation were only available for 55 patients. In addition, MRI scans were not available in 6 of these cases and CT scans were utilised instead. Whilst a prospective study would allow for imaging protocols to be more unified, due to the rare nature of this condition and the ˜40-year span of the study, imaging quality and even modalities would likely change over this period. Future studies would benefit from multi-centre collaboration to boost sample sizes. Another implication of using historical data is the incomplete nature of the clinical information. We were only able to identify cluster locations in three-quarters of the patients with encephaloceles. Furthermore, information regarding any underlying genetic abnormalities such as Meckel-Gruber syndrome or other chromosomal abnormalities was also not recorded in our data capture.

We are also unable to estimate the potential effect of selection bias on our data. It may be that encephaloceles do occur at different locations in the embryo compared to those that we have identified from post-natal imaging, but these may not be compatible with life resulting in early miscarriage^34^. Whilst this information is not currently available, the utilisation of early prenatal identification of encephaloceles raises the possibility of this in the future^35^. Our database includes only patients who underwent surgical treatment of the encephalocele, and it is possible that a proportion of patients may have had such severe defects that closure was not appropriate or possible; some parents may have declined surgical intervention. At the opposite end of the spectrum, it is also possible that a proportion of patients have very small atretic encephaloceles that never come to neurosurgical attention.

Another limitation is the challenge associated with estimating the topographical origin of the skull defect from post-natal imaging, especially with large defects. In the development of the atlas, we applied two approaches to estimate the origin of the defect. The first was to overlap all the segmented bone defects from patients with volumetric imaging following non-linear transformation into template space to create a heat map. The use of an age-appropriate template allows for the correction of the varying degrees of microcephaly as well as distortions in skull anatomy. The corrections applied to the skull and consequently to the skull defect, brain herniation and encephalocele volume allow for objective and systematic comparison and co-localisation of the defect. The second approach was to apply a clustering analysis. Whilst this method has not been used previously to study encephaloceles it has been used for a wide range of machine learning applications and in healthcare^36,37^. Broadly speaking clustering techniques can be separated into agglomerative or hierarchical methods. In this study, we applied the agglomerative K-means clustering method, although similar results were also achieved through a hierarchical method (see dendrogram in supplementary material). For the K-means clustering technique, a single point is needed to represent the encephalocele origin instead of the entire skull defect. As we do not know where the skull defect originated within the embryo, we estimated this through calculation of the morphological centre of the segmented skull defect. We acknowledge that this is maybe imprecise for larger defects. To mitigate this, we also developed a heat map based on the overlap of the bone defect segmentation and identified similar results. We refer the reader to the supplementary material for further explanation of the clustering method.

## 5 Conclusion

We present a large series of patients undergoing surgical treatment of encephalocele over ˜40 years. Utilising volumetric imaging we present a heat map and topographical atlas of encephalocele locations. The application of a K-means clustering algorithm identified encephaloceles arise in three locations: 1) Anterior skull base 2) Parieto-occipital junction and 3) Peri-torcular. The parieto-occipital junction location was most common in our series and was most likely to be associated with a falcine sinus and least likely to have brain contents within the encephalocele. Despite there being no difference in the size of the bone defects between the different clusters, peri-torcular encephaloceles were the largest in volume. The data presented here provide correlative human data to novel animal models of encephalocele that point towards a post-neurulation aetiology.

## Supplementary Material

Suppmementary material

## Figures and Tables

**Table 1:** Ethnicity assigned based on self-declared ethnicity from hospital records

Assigned ethnicity	Declared ethnicity
White	English Welsh Scottish Northern Irish British Irish Gypsy or Irish Traveller Any other White background
Asian / Asian British	Indian Pakistani Bangladeshi Chinese Any other Asian background
Black / Black British	African Caribbean Any other Black, African or Caribbean background
Other ethnic groups	Arab Any other ethnic group

**Table 2:** Baseline Demographics

Variable	Parameter	Parieto-occipital junction	Peri-torcular	Anterior skull base	Not documented	Total
Gender	Male	33% (17/52)	27% (14/52)	25% (13/52)	15% (8/52)	43% (52/122)
	Female	21% (15/70)	36% (25/70)	11% (8/70)	31% (22/70)	57% (70/122)
Ethnicity	White	12	18	10	21	50% (61/122)
	Black	5	12	4	0	17% (21/122)
	Asian	7	6	2	4	16% (19/122)
	Other	8	1	4	3	13% (16/122)
	Undisclosed	0	2	1	2	4% (5/122)
Encephalocele	Presence of Brain herniation	17% (4/24)	70% (14/20)	73% (8/11)	-	47% (26/55)
	Venous abnormality	67% (16/24)	50% (10/20)	27% (3/11)	-	53% (29/55)

**Table 3:** Contingency table of actual and expected values for the ethnicity based on the cluster assignment

	Ethnicities - Actual (Expected)				
Cluster assignment	Asian	Black	Other	White	Sum
Anterior skull base	2 (2.3)	4 (1.0)	4 (0.3)	10 (26.1)	20
Parieto-occipital	7 (2.3)	5 (1.0)	8 (0.3)	12 (26.1)	32
Peri-torcular	6 (2.3)	12 (1)	1 (0.3)	18 (26.1)	37

## References

[1] Wen S, Ethen M, Langlois PH, Mitchell LE (2007). Prevalence of encephalocele in Texas, 1999-2002. Am J Med Genet Part A.

[2] Peake JN, Knowles RL, Shawe J, Rankin J, Copp AJ (2021). Maternal ethnicity and the prevalence of British pregnancies affected by neural tube defects. Birth defects Res.

[3] Siffel C, Wong L-YC, Olney RS, Correa A (2003). Survival of infants diagnosed with encephalocele in Atlanta, 1979-98. Paediatr Perinat Epidemiol.

[4] Parker SE, Mai CT, Canfield MA (2010). Updated National Birth Prevalence estimates for selected birth defects in the United States, 2004-2006. Birth Defects Res A Clin Mol Teratol.

[5] Copp AJ, Stanier P, Greene NDE (2013). Neural tube defects: recent advances, unsolved questions, and controversies. Lancet Neurol.

[6] Fisher NL, Smith DW (1981). Occipital encephalocele and early gestational hyperthermia. Pediatrics.

[7] Dutta HK, Deori P (2010). Anterior encephaloceles in children of Assamese tea workers Clinical article. J Neurosurg Pediatr.

[8] Monteith SJ, Heppner PA, Law AJJ (2005). Encephalocoele-- epidemiological variance in New Zealand. J Clin Neurosci Off J Neurosurg Soc Australas.

[9] Da Silva SL, Jeelani Y, Dang H, Krieger MD, McComb JG (2015). Risk factors for hydrocephalus and neurological deficit in children born with an encephalocele. J Neurosurg Pediatr.

[10] Yucetas SC, Uçler N (2017). A Retrospective Analysis of Neonatal Encephalocele Predisposing Factors and Outcomes. Pediatr Neurosurg.

[11] Markovic I, Bosnjakovic P, Milenkovic Z (2020). Occipital Encephalocele: Cause, Incidence, Neuroimaging and Surgical Management. Curr Pediatr Rev.

[12] Cavalheiro S, da Costa Silva MD, Nicácio JM (2020). Fetal surgery for occipital encephalocele. J Neurosurg Pediatr.

[13] Teixeira AAR, de Melo Neto FF, de Abreu NMP, Dias DA, Souza MLP (2020). Anatomical implications of posterior cephaloceles in the dural venous sinuses. Child’s Nerv Syst ChNS Off J Int Soc Pediatr Neurosurg.

[14] von Recklinghausen F (2011). The classic: Studies on spina bifida. 1886. Clin Orthop Relat Res.

[15] Padmanabhan R (2006). Etiology, pathogenesis and prevention of neural tube defects. Congenit Anom (Kyoto).

[16] Rolo A, Galea GL, Savery D, Greene NDE, Andrew J (2019). Novel mouse model of encephalocele: Post-neurulation origin and relationship to open neural tube defects. DMM Dis Model Mech.

[17] Rowland CA, Correa A, Cragan JD, Alverson CJ (2006). Are encephaloceles neural tube defects?. Pediatrics.

[18] Fonov V, Evans AC, Botteron K, Almli CR, McKinstry RC, Collins DL (2011). Unbiased average age-appropriate atlases for pediatric studies. Neuroimage.

[19] Sammouda R, El-Zaart A (2021). An Optimized Approach for Prostate Image Segmentation Using K-Means Clustering Algorithm with Elbow Method. Comput Intell Neurosci.

[20] Kotil K, Kilinc B, Bilge T (2008). Diagnosis and Management of Large Occipitocervical Cephaloceles: A 10-Year Experience. Pediatr Neurosurg.

[21] Kıymaz N, Yılmaz N, Demir İ, Keskin S (2010). Prognostic Factors in Patients with Occipital Encephalocele. Pediatr Neurosurg.

[22] Suwanwela C, Suwanwela N (1972). A morphological classification of sincipital encephalomeningoceles. J Neurosurg.

[23] Komotar RJ, Starke RM, Raper DMS, Anand VK, Schwartz TH (2013). Endoscopic endonasal versus open repair of anterior skull base CSF leak, meningocele, and encephalocele: a systematic review of outcomes. J Neurol Surg A Cent Eur Neurosurg.

[24] Recklinghausen Fv (1886). Ueber die Art und die Entstehung der Spina bifida, ihre Beziehung zur Rückenmarks-und Darmspalte. Arch für Pathol Anat und Physiol und für Klin Med.

[25] Schwartz JS, Adappa ND, Lal D, Hwang PH (2019). Frontal Sinus Surgery: A Systematic Approach.

[26] O’rahilly R, Müller F (1989). Bidirectional closure of the rostral neuropore in the human embryo. Am J Anat.

[27] Müller F, O’Rahilly R (1991). Development of anencephaly and its variants. Am J Anat.

[28] Sharma S, Ojha BK, Chandra A, Singh SK, Srivastava C (2016). Parietal and occipital encephalocele in same child: A rarest variety of double encephalocele. Eur J Paediatr Neurol EJPN Off J Eur Paediatr Neurol Soc.

[29] Canaz H, Ayçiçek E, Akçetin MA, Akdemir O, Alataş I, Özdemir B (2015). Supra- and infra-torcular double occipital encephalocele. Neurocirugia (Astur).

[30] Singh DK, Singh N, Kumar P (2012). Double suboccipital meningoencephalocele: a unique case report. Pediatr Neurosurg.

[31] McBratney-Owen B, Iseki S, Bamforth SD, Olsen BR, Morriss-Kay GM (2008). Development and tissue origins of the mammalian cranial base. Dev Biol.

[32] Sencer S, Arnaout MM, Al-Jehani H, Alsubaihawi ZA, Al-Sharshahi ZF, Hoz SS (2021). The spectrum of venous anomalies associated with atretic parietal cephaloceles: A literature review. Surg Neurol Int.

[33] McBain L, Goren O, Shane Tubbs R (2020). Tubbs Imaging and Surgery of the Intracranial Dural Venous Sinuses RSBT-A.

[34] Creasy MR, Alberman ED (1976). Congenital malformations of the central nervous system in spontaneous abortions. J Med Genet.

[35] Tonni G, Ventura A, Bonasoni MP (2009). Acrania/encephalocele sequence (exencephaly) associated with 92,XXXX karyotype: early prenatal diagnosis at 9(+5) weeks by 3D transvaginal ultrasound and coelocentesis. Congenit Anom (Kyoto).

[36] Steinley D (2006). K-means clustering: a half-century synthesis. Br J Math Stat Psychol.

[37] Dubey AK, Gupta U, Jain S (2016). Analysis of k-means clustering approach on the breast cancer Wisconsin dataset. Int J Comput Assist Radiol Surg.

