# Supplementary material for: A Topographical Analysis of Encephalocele Locations: Generation of a Standardised Atlas and Cluster Analysis
Source: Childs Nerv Syst. Author manuscript; Available in PMC 2023 Jul 1. (PMC7614697; doi:10.1007/s00381-023-05883-7)
Supplement: Suppmementary material [file EMS173363-supplement-Suppmementary_material.docx]

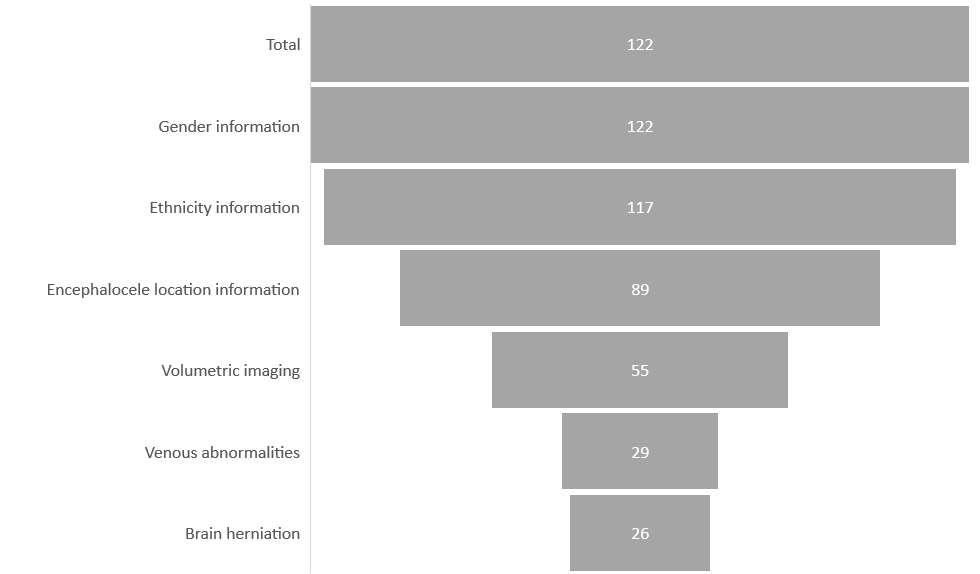


**Supplementary Figure 1. Available Data From Encephalocele Case Series.** Pyramidal bar chart depicting the number of patients for which information was available or the presence of specific findings (venous abnormalities and brain herniation) in relation to the entire cohort. As described in the methods section, the location of the centroids of the bone defects associated with each encephalocele could be resolved by the Y- and Z-dimension coordinates. Clustering methods are used to group these locations based on their position. Two common methods include K-means and Hierarchical clustering. An explanation of how K-means clustering functions is shown in Supplementary Figure 2.

Due to the retrospective nature of the case series spanning almost 40 years complete information or data were not available in all cases. Furthermore the development in imaging paradigms and availability has led to heterogeneity within the data. Supplementary Figure 1 depicts the number patients for which data is available for the different analyses.


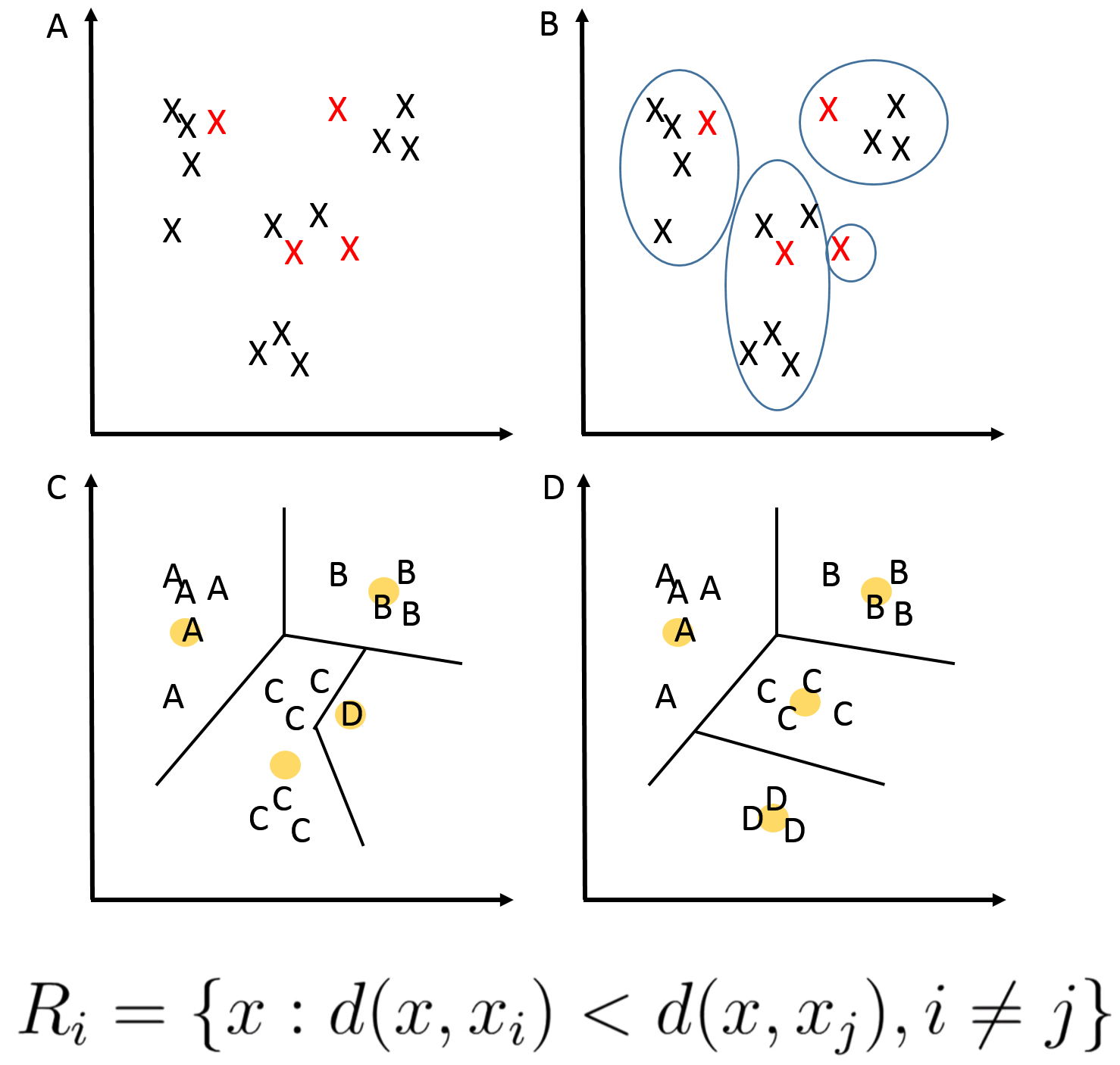


**Supplementary Figure 2. Example of K-means clustering.** Data points are resolved by their coordinates. A) The K-means clustering method randomly assigns K data points as starting nodes. As an example, we assign K=4 and these are represented by a red X. B) Based on these starting locations, the closest data points based on the Euclidean distance are then included within the cluster (blue circles). C) Based on all data points within the clusters, the cluster centroids are then calculated (Yellow dot). D) The data points are then re-assigned based on the Euclidean distance. Steps C and D are repeated until no more re-assignment takes place. The black lines represent the ascribed Voronoi partitions between clusters. The equation describing the assignment of clusters is shown at the bottom of the figures.

Another method of visualising the clustering is through a hierarchical clustering method in the form of a dendrogram (Supplementary Figure 3). The dendrogram functions by taking each data point and linking to the next closest data point. This is then repeated until clusters form. To determine the optimal number of clusters for the K-means clustering (K) we applied the elbow method. The dendrogram is another method that can also be used to determine K by identifying the fewest clusters resulting in the lowest Euclidean distance between them. This confirms K=3.


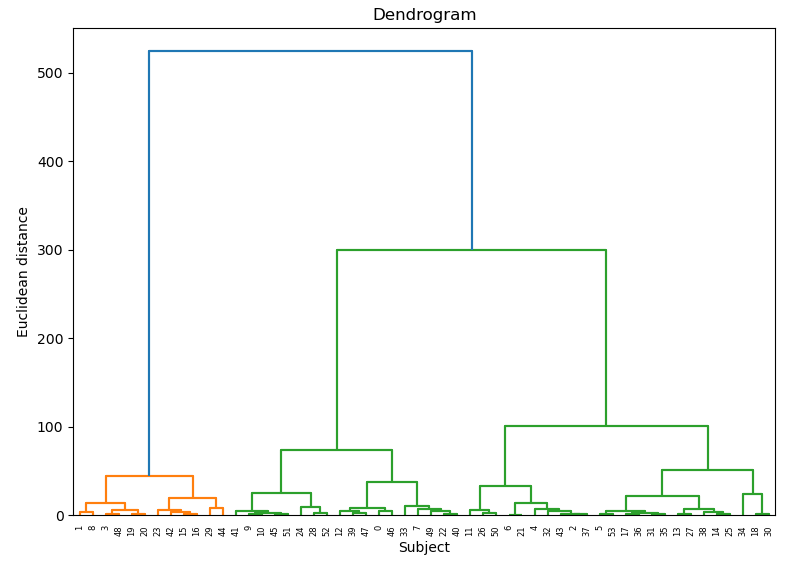


**Supplementary Figure 3. Dendrogram of Bone Defect Centroids.** Dendrogram representing a hierarchical clustering method applied to each subject within the cohort. The height of the linkages (length of the vertical lines) represents the Euclidean distance between the individual or clustered data points.
